# Supplementary material for: Isorhamnetin in Quinoa Whole-Grain Flavonoids Intervenes in Non-Alcoholic Fatty Liver Disease by Modulating Bile Acid Metabolism through Regulation of FXR Expression
Source: Foods. 2024 Sep 26;13(19):3076. doi: 10.3390/foods13193076 (PMC11475887; doi:10.3390/foods13193076)
Supplement: Supplementary file 1 [file foods-13-03076-s001.zip › foods-3207508-supplementary.pdf]

Figure S1

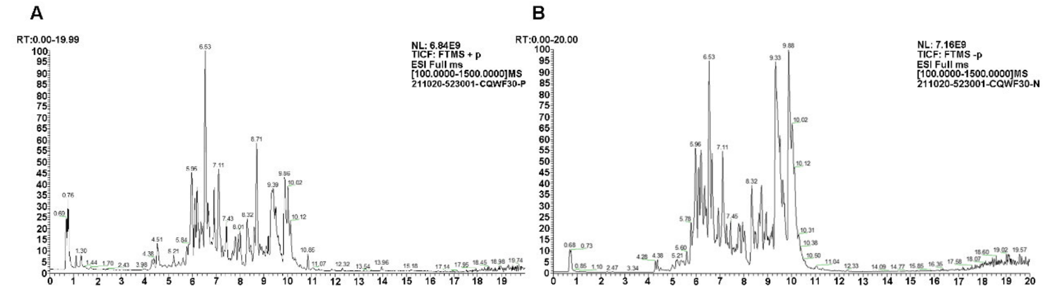

Figure S1. CQWF30 mass spectrometry results. Total ion chromatogram (TIC) in positive ion mode for the CQWF30 sample (A). TIC in negative ion mode for the CQWF30 sample (B).

Table S1 Liquid chromatography mobile phase conditions

Time (min)	Flow rate (μL/min)	Gradient	B% Acetonitrile
0-2	300	-	5
2-6	300	Linear gradient	30
6-7	300	-	30
7-12	300	Linear gradient	78
12-14	300	-	78
14-17	300	Linear gradient	95
17-20	300	-	95
20-21	300	Linear gradient	5
21-25	300	-	5

Table S2 The flavonoids composition in CQWF30.

No.	RT [min]	Formula	Class of compound	Name	[M+H]+/[M-H]-	MW	Percentage of CQWF30
1	6.20	$C_{33}H_{40}O_{19}$	Flavanoids	Mauritianin	739.21	740	14.56
2	6.68	$C_{15}H_{10}O_7$	Flavonoids	Quercetin	301.04	302	10.54
3	6.11	$C_{32}H_{38}O_{20}$	Flavanoids	Helicianeoide B	741.19	742	9.28
4	6.23	$C_{15}H_{10}O_6$	Flavonoids	Kaempferol	287.23	286	8.83
5	6.70	$C_{21}H_{20}O_{12}$	Flavanoids	Isoquercitrin	463.09	464	8.66
6	6.82	$C_{21}H_{18}O_{13}$	Flavanoids	Quercetin3-*O*-β-D-Glucuronide	477.07	478	7.9
7	6.27	$C_{27}H_{30}O_{15}$	Flavanoids	Nictoflorin	595.52	594	7
8	6.05	$C_{27}H_{30}O_{16}$	Flavanoids	Quercetin3-*O*-glucoside-7-*O*-rhamnoside	609.15	610	6.79
9	6.47	$C_{27}H_{30}O_{16}$	Flavonoids	Rutin	611.52	610	3.84
10	6.29	$C_{34}H_{42}O_{20}$	Flavonoids	Typhaneoside	771.69	770	3.43
11	7.07	$C_{21}H_{18}O_{12}$	Flavanoids	Scutellarin	463.37	462	2.73
12	7.07	$C_{21}H_{18}O_{12}$	Flavanoids	Kaempferol-3-glucuronide	461.07	462	2.41
13	6.36	$C_{32}H_{38}O_{19}$	Flavanoids	Camelliaside B	725.19	726	2.32

No.	RT [min]	Formula	Class of compound	Name	$[M+H]^+/[M-H]^-$	MW	Percentage of CQWF30
14	8.04	$C_{14}H_{22}O$	Flavanoids	4-Octylphenol	205.46	206	2.22
15	6.37	$C_{26}H_{28}O_{16}$	Flavanoids	Quercetin 3-*O*-β-D-ribosyl-(1-2)-β-D-glucoside	595.13	596	2.18
16	6.01	$C_{21}H_{20}O_{12}$	Flavanoids	Hyperoside	465.38	464	2.15
17	6.25	$C_{21}H_{20}O_{11}$	Flavanoids	Orientin	449.38	448	1.91
18	6.52	$C_{28}H_{32}O_{16}$	Flavonoids	Narcissin	623.16	624	1.34
19	6.30	$C_{16}H_{12}O_7$	Flavanoids	Isorhamnetin	317.26	316	0.82
20	6.88	$C_{28}H_{32}O_{16}$	Flavonoids	Isoscoparin-2"-β-D-glucopyranoside	623.16	624	0.71
21	7.12	$C_{22}H_{22}O_{12}$	Flavanoids	Isorhamnetin3-galactoside	477.10	478	0.24
22	2.97	$C_8H_7NO_2$	Flavanoids	4-Hydroxymandelonitrile	150.15	149	0.08
23	9.13	$C_{16}H_{12}O_5$	Flavanoids	Glycitein	283.06	284	0.08
24	5.23	$C_{27}H_{30}O_{17}$	Flavanoids	Baimaside	625.14	626	0.05
25	9.13	$C_{16}H_{12}O_5$	Flavanoids	5-*O*-Methylgenistein	285.26	284	0.01

2.3 UPLC-ESI-MS/MS Experiment

2.3.1 Positive Mode: Heater Temperature 325°C; Sheath Gas Flow Rate: 45 arb; Auxiliary Gas Flow Rate: 15 arb; Purging Gas Flow Rate: 1 arb; Electrospray Voltage: 3.5 KV; Capillary Temperature: 330 °C; S-Lens RF Level: 55%.

2.3.2 Negative Mode: Heater Temperature 325 °C; Sheath Gas Flow Rate: 45 arb; Auxiliary Gas Flow Rate: 15 arb; Purge Gas Flow Rate: 1 arb; Electrospray Voltage: 3.5 KV; Capillary Temperature: 330 °C; S-Lens RF Level: 55%.

2.3.3 Scanning modes: Full Scan (m/z 100~1500) with data-dependent secondary mass spectrometry (dd-MS2, TopN = 10); Resolution: 120,000 (primary mass spectrometry) & 60,000 (secondary mass spectrometry). Collision mode: High energy collisional dissociation (HCD).

2.3.4 The components in CQWF30 were subsequently identified by primary MS and 25 potentially bioactive flavonoids in CQWF30 were further identified by secondary MS. The following table shows the 25 active flavonoids identified by MS in CQWF30.

2.10.5.1 Identification of bile acids by LC-MS/MS Experiment

Instruments, reagents and sample preparation

The main instruments used in this experiment were an LC-20AD HPLC (Shimadzu Corporation) and an ABSCIEX4000Q TRAP mass spectrometer (Scientific Export).

In addition, a total of 20 bile acid controls were available in this experiment for parametric comparison of the identified bile acids.

Take mouse liver tissue, add activated charcoal to remove endogenous bile acids, homogenize and stir, centrifuge, and take the supernatant as the blank matrix. The rest of the tissue was homogenized, centrifuged and the supernatant was taken. 50 μL was sucked up precisely, 200 μL of the internal standard solution was added, vortexed for 10 min, centrifuged at 12 000 r/min for 10 min, 200 μL of the supernatant was taken, blown dry under nitrogen gas, reconstituted with 100 μL of methanol, vortexed for 3 min, and then injected into the sample for analysis.

Mass Spectrometry Conditions

The separation was performed on a Waters Symmetry C18 column (2.1 mm×150 mm, 3.5 μm) with the mobile phase A as an aqueous solution containing 0.1% formic acid and 10 mmol/L ammonium acetate, and the mobile phase B as a methanol solution containing 0.1% formic acid and 10 mmol/L ammonium acetate, and the gradient elution (0~2 min, 60% B; 2~40 min. 60%B→90%B; 40~45 min, 90%B; 45~50 min, 60%B) at a flow rate of 0.15 mL/min with an injection volume of 10 μL, column temperature of 40℃, and autosampler: 4℃.

The mass spectrometry conditions were electrospray ionization (ESI), negative ion mode (-), and multiple ion reaction monitoring (MRM). The working parameters were as follows: air curtain gas was 137.9 kPa; ion source voltage was -4.5 kV; ion source temperature was 300℃; GS1 was 275.8 kPa; GS2 was 275.8 kPa; Interface Heater: on; EP was -10 V; and CXP was -13 V. The ESI was operated in the negative ion mode (-), and MRM was performed in the negative ion mode (-).

Each bile acid control was weighed precisely, placed in a 100 mL measuring flask, and dissolved with methanol to configure the standard control. The internal standard d4-GCDCA was weighed precisely, dissolved with acetonitrile, fixed, and prepared into a solution with a concentration of 5.0 μmol/L (acetonitrile was used as a protein precipitant).

The sample was analyzed by the mass spectrometer, and the bile acids detected were as shown in Table 2.
